# To the Editors of the Medical and Physical Journal

**Published:** 1801-09

**Authors:** 


### *To the Editors of the Medical and Phyſical Journal.*

GENTLEMEN,

IN the laſt number of the Medical and Phyſical Journal, you ſay in a note, p. 121, that "when the Vaccine virus is taken after the areola is fully formed, it is not to be depended upon as a preventive of the Small-pox, although it will produce a diſeaſe imitative of the true Vaccine."

Perhaps this ſentence may not appear perfectly intelligible to other practitioners any more than it does to me. I would therefore wiſh to enquire, whether the virus taken after the full formation of the areola *never* produces the genuine Cow-pox, or whether it only *sometimes* fails; and, in this caſe, by what characteriſtics the imitative diſeaſe is to be diſtinguiſhed from the genuine?

I am anxious to procure information on this ſubject, as I have lately, from circumſtances, been induced to inoculate a patient with virus taken on the eleventh day after the inciſion, and after after the full formation of the areola. This inoculation produced a difeafe in every refpect fo fimilar to the genuine vaccine, that I was perfectly fatisfied of my patient's fecurity from variolous infection, till the above note raifed fome doubts in my mind, which I hope you will have the goodnefs to difpel.

I can recollect no variance whatever from the genuine difeafe, of which I have feen a very fufficient number of cafes, unlefs the feparation of the fcab fooner than ufual is to be reckoned one. The patient, a child about eight weeks old, was inoculated on the 1ft of the month, and the fcab came off with the flighteft poffible force on the 17th. In every other refpect the appearance was ftrongly characteriftic of the Cow-pox, the infection, inflammation, and every fymptom took place in the moft regular and fatisfactory manner.

Your attention to this note in your next number, will confer an obligation on, Gentlemen, your conftant reader.

M.

Our defign in the Note above alluded to, was to inculcate the advice fo often given by Dr. Jenner, to ufe none but early virus; and alfo to inform our readers that matter taken after the areola was fully formed had produced a difeafe, not at prefent to be diftinguifhed from the true Vacciola by any defcription, but which failed to prevent the fmall-pox. We do not believe that it will always fail, but we think it ought never to be trufted. Ingenious artifts are now at work, in the hope of being able to give accurate reprefentations of the true and fpurious puftules.

In the mean time, why fhould practitioners expofe themfelves and patients to hazard, when early virus can be fo eafily procured? EDIT.

---

