# Errata

**Published:** 1801-12

**Authors:** 


### ERRATA.

Page 218, l. 20, to W. B. Bayley, *add* M. D.

